# The Night Effect of Anger: Relationship with Nocturnal Blood Pressure Dipping

**DOI:** 10.3390/ijerph17082705

**Published:** 2020-04-15

**Authors:** Maria Casagrande, Francesca Favieri, Angela Guarino, Enrico Di Pace, Viviana Langher, Giuseppe Germanò, Giuseppe Forte

**Affiliations:** 1 Dipartimento di Psicologia Dinamica e Clinica–Università di Roma “Sapienza”, Via degli Apuli 1, 00185 Roma, Italy; viviana.langher@uniroma1.it; 2 Dipartimento di Psicologia—Università di Roma “Sapienza”, Via dei Marsi 78, 00185 Roma, Italy; francesca.favieri@uniroma1.it (F.F.); angela.guarino@uniroma1.it (A.G.); enrico.dipace@uniroma1.it (E.D.P.); 3 Dipartimento di Scienze Cardiovascolari, Respiratorie, Nefrologiche e Geriatriche–Università di Roma “Sapienza”, Piazzale Aldo Moro, 00185 Roma, Italy; ncgerman@tin.it

**Keywords:** blood pressure, dipping status, anger, ambulatory blood pressure monitor

## Abstract

Introduction: The circadian pattern of blood pressure is characterized by a physiological drop occurring after sleep onset. The alteration of this phenomenon (non-dipping, extreme dipping, or reverse dipping) is associated with an increased cardiovascular risk. Besides altered autonomic and endocrine circadian rhythms, psychological aspects seem to play a role in this modification. However, the few studies that have analyzed the influence of psychological dimensions on the dipping phenomenon have reported inconsistent results. This study aimed to examine the relationship between anger expression and blood pressure (BP) dipping. Methods: We obtained 24 h ambulatory BP measurements from 151 participants and used them to define three groups according to their dipping status: Dippers (*N* = 65), Non-Dippers (*N* = 42), and Extreme Dippers (*N* = 44). Sociodemographic and anamnestic information was collected, and the State–Trait Anger Expression Inventory was used to assess anger. Results: Analysis of variance evidenced significant higher scores for Trait Anger Temperament and Anger Expression in Extreme Dippers than in both Dippers and Non-Dippers. However, after controlling for confounding variables, there was no significant relationship with trait anger, and only the result concerning the suppression of anger was confirmed. Conclusions: These findings suggest that the analysis of some psychological factors, such as anger, could be necessary to better understand differences in nocturnal BP alterations. Trait anger and suppression of anger may contribute to the description and classification of patients who exhibit a maladaptive dipping phenomenon. However, modifiable (i.e., cigarette consumption) and unmodifiable (i.e., age) risk factors appear to mediate this relationship. Although further studies are necessary to explore this association, these results highlight that some aspects of anger can represent risk factors or markers of maladaptive modulation of the dipping phenomenon.

## 1. Introduction

Blood pressure (BP) follows a reproducible circadian pattern characterized by 10%–20% drops or “dips” after sleep onset [1,2,3]. This physiological phenomenon is commonly indicated as “dipping” and appears to be protective for cardiovascular health [4]. Dipping is mostly due to endogenous circadian rhythms of the autonomic nervous and endocrine systems, as well as to exogenous patterns of activity during the 24 h [5,6]. Sometimes, the nocturnal blood pressure trend evidences some alterations, and people do not present BP dipping. Besides the absence of this status (Non-Dipping), different declinations of BP dipping may occur. In some individuals, an excessive reduction of BP (Extreme Dipping), or an increase of the BP trend after sleep onset (Reverse Dipping) may be reported. Earlier studies mainly focused on the absence of dipping, highlighting that a non-dipping pattern is a significant predictor of a range of organ damage and cardiovascular events [3,7,8]. The increase of cardiovascular risk associated with Non-Dipping seems to be due to the difficulty in physiological recovering from the typical demand-driven elevations of blood pressure during the waking hours of the day [9] and it is characterized by a disruption of the circadian rhythm of the autonomic balance [10]. Accordingly, some studies suggested that Non-Dippers have a high sympathetic nervous system activity during wake time [11,12,13] and a lower parasympathetic nervous system activity throughout the sleep period [13,14].

However, the exact causes of Non-Dipping are not fully clarified [15,16,17], and genetic, biological, environmental, and sociodemographic factors could affect it [18,19]. An increase in sympathetic nervous system activity appears to be associated with some psychological states [20,21] and cognitive dysfunctions [22,23]. It is well known that some psychological factors, such as hostility, depression, anxiety, dysfunctional coping strategies, and emotional dysregulation, appear to have a role in both cardiovascular events and the development and maintenance of hypertension [24,25,26,27,28,29]. A growing body of evidence suggests that also the variations in nocturnal BP dipping are influenced by some psychosocial factors, such as anger and hostility [30,31], perceived racism/discrimination [32], job strain [33], depression [34], and social support [35].

Many studies focused their attention on the relationship between BP dipping status and hostility and anger [30,31,36]. Within these studies, those that examined the association of dipping status with anger and anger management styles obtained mixed results [16,17,36] and failed to clarify the psychophysiological relationship between anger and the dipping phenomenon.

The present study aimed to investigate the association between anger and BP dipping status in both healthy adults and people with essential hypertension, without other medical conditions. Moreover, unlike previous studies focused on the predictive role of anger in dipping ratio variations, we aimed to analyze the characteristics of anger in people with different dipping patterns. Therefore, we compared Dippers, non-Dippers, and Extreme Dippers. In the analyses of the relationship between the BP dipping and anger, we also considered the effect of some confounding variables, such as age, body mass index, smoking, and alcohol intake.

The hypothesis that the dipping pattern is linked to anger is supported by other studies [30,31] and by researches that considered the Non-Dipping phenomenon as a major health risk factor [3,7,8]. According to these studies, we hypothesized that higher traits and expressions of anger are present in individuals with a Non-Dipping status; furthermore, we expected higher levels of anger in people with an Extreme-Dipping status compared to those with a physiological BP dipping status.

## 2. Method

### 2.1. Participants

One-hundred and fifty-one participants were recruited at the First Medical Clinic of the Policlinico Umberto I at the University of Rome “Sapienza”. A cardiologist supplied information about the diagnosis of hypertension and the circadian blood pressure pattern. The participants were divided into three groups: Dippers (*N* = 65 [29 Men, 36 Women]; Age = 56.08 years; SD = 10.38), who had a night/day BP ratio between 0.80 and 0.90; Non-Dippers (*N* = 42 [20 Men, 22 Women]; Age = 59.86 years; SD = 8.74), who presented a ratio higher than 0.90; and Extreme Dippers (*N* = 44 [18 Men, 26 Women]; Age = 54.79 years; SD = 8.76), who showed a ratio lower than 0.80.

Inclusion criteria were: age between 40 and 75 years, to better control autonomic changes related to age that could affect this relationship, absence of chronic medical conditions, such as cancer, diabetes, and cardiac, neurological, and psychiatric disorders.

### 2.2. Assessment Tools

#### 2.2.1. Physiological Measures

Systolic (SBP) and Diastolic Blood Pressure (DBP) were recorded by using an automatic electronic sphygmomanometer validated for self-measurement (“Personal Check” PIC) [37]. Blood pressure measurement was performed according to the European Guidelines for Hypertension [38].

A balance and a meter were used to measure the weight and height of the participants and calculate the Body Mass Index (BMI: kg/m^2^) [39].

#### 2.2.2. Ambulatory Blood Pressure Monitor (ABPM)

The 24 h BP was measured with the Takeda ABPM monitor (TM-2430). The ABPM was set to obtain BP recordings at 15 min intervals during the day (07:00 to 22:00) and 30 min intervals during the night (22:00 to 07:00). However, we choose to consider as “night” an interval comprised from 00:00 to 06:00, in line with other studies [40]. No “out-of-bed" readings at night were included. This information was recorded and transferred to the computer analysis system. Initially, the ABPM recording of each person was automatically scrutinized to edit out artifactual recordings. The predetermined editing criteria were an SBP greater than 240 mmHg or lower than 70 mmHg or a DBP higher than 150 mmHg or lower than 40 mmHg. Additionally, to be included in the analysis, each ABPM dataset had to include a minimum of two-thirds of the SBP and DBP measurements during both the daytime and the night-time periods. These criteria followed the practice guidelines for ambulatory BP measurement [40].

The ABPM sessions were performed during a weekday, and participants were instructed to attend their usual daily activities but to hold their arm stationary during BP readings. Additionally, they filled in a log on the activities carried out during the BP registration day and they reported the time of both sleep onset and awakening. Furthermore, they recorded whether they were out of bed during the night (e.g., to use the bathroom) and the relative awake time.

The ABPM recordings allow calculating the Mean Arterial Pressure (MAP) of wake and sleep periods through the formula MAP = [SBP + (2 × DBP)]/3 [41,42].

In the present study, BP dipping was defined as a reduction between 10% and 20% in the MAP from day to night, Non-Dipping BP was defined as a reduction in MAP from day to night lower than 10%, and Extreme Dipping BP was defined as reduction higher than 20% of the mean MAP from day to night [41].

#### 2.2.3. Socio-Demographic and Anamnestic Information

Demographic data (age, gender, years of education), lifestyles (smoking and alcohol consumption), medical and psychiatric information were collected for each participant by face-to-face interview.

#### 2.2.4. Spielberger State–Trait Anger Expression Inventory (STAXI)

The STAXI [43,44] is a self-report questionnaire that allows assessing different dimensions of anger. It includes 44 items, divided into three sections (State Anger, Trait Anger, and Anger Expression/Control). The responses to the items are given by using a 4-point Likert scale. State Anger (S-Anger) indicates the anger experienced by the participant when completing the questionnaire and includes 10 items. Trait anger (T-Anger) is measured by 10 items requiring participants to report how frequently angry feelings are experienced over time; it includes two subscales: Trait Anger Temperament (T-Anger.T) and Trait Anger Reaction (T-Anger.R), both composed of four of the items of the T-Anger section. The Anger Expression/Control section includes four different subscales (Anger expression-in, Anger expression-out, Anger control-in, Anger control-out). Anger Expression-In (Anger.E-In; 8 items) assesses how often angry feelings are experienced but suppressed; Anger Expression-Out (Anger.E-Out; 8 items) measures how often anger is expressed in physical or verbal aggression; Anger Control-In (Anger.C-In; 8 items) assesses how often a person attempts to control angry feelings by actively calming oneself. These three subscales of the Anger Expression/Control section contribute to determining the Anger Control-Out (Anger.C-Out), which assesses how frequently a person attempts to control the outward expression of angry feelings.

## 3. Procedure

All participants gave their informed consent for inclusion in the research. The study was conducted according to the Declaration of Helsinki, and the Local Ethics Committee (Department of Dynamic and Clinical Psychology—“Sapienza” University of Rome; number: 0001166, approved on 30 July 2019) approved the protocol. After the participants had signed informed consent, they were subjected to blood pressure recordings; then, weight and height were measured. Finally, the participants completed the socio-demographic and anamnestic interview and the STAXI questionnaire. After this procedure, a cardiologist gave the eventual diagnosis of essential hypertension and classified the dipping status subsequently to the analysis of the Holter data. The whole procedure, lasting about 40 min, took place in a quiet environment with a comfortable temperature.

### Statistical Analyses

One-way analyses of variance (ANOVAs) were carried out considering the Group as the independent variable (Dippers, Non-Dippers, Extreme Dippers) and the different socio-demographic (age, years of education), physiological (SBP, DBP, MAP day, MAP night, night/day MAP ratio, BMI), and lifestyles (smoking and alcohol consumption) dimensions as dependent variables.

ANOVAs considering as outcomes the STAXI subscales were conducted. Age, gender, years of education, body mass index, smoking, and alcohol consumption were examined as potential confounders in data analysis. Planned comparisons were used to analyze significant effects.

The Chi Squared test (χ^2^) was used to estimate Non-Dippers, Dippers, and Extreme Dippers in the three groups of Normotensive, Untreated, and Treated Hypertensive participants.

To assess the percentage of hypertensive patients in Non-Dippers, Dippers, and Extreme Dippers and to verify the differences in proportion between men and women in the three groups of participants, the χ^2^ test was used.

Correlations among variables were evaluated by using Pearson’s r coefficient.

For all the statistical analyses, the level of significance was accepted at *p* < 0.05. Statistical analyses were performed through the Statistica Software v.10.0.

## 4. Results

### 4.1. Demographical, Lifestyles, and Physiological Variables

The main characteristics of the participants are shown in Table 1.

The ANOVA on age showed significant differences between groups (F_2,148_ = 3.35; *p* = 0.04; *pƞ*^2^ = 0.04); Non-Dippers were older than both Dippers (F_1,148_ = 4.05; *p* = 0.05; *pƞ*^2^ = 0.03) and Extreme Dippers (F_1,148_ = 6.11; *p* = 0.01; *pƞ*^2^ = 0.04). No differences were highlighted between Dippers and Extreme Dippers (F_2,148_ = 0.48; *p* = 0.48).

The ANOVA on the cigarette consumption showed a significant difference between groups (F_2,148_ = 3.10; *p* = 0.05; *pƞ*^2^ = 0.04); Dippers had higher consumption of cigarettes than Non-Dippers (F_1,148_ = 4.97; *p* = 0.03; *pƞ*^2^ = 0.03) and marginally than Extreme Dippers (F_1,148_ = 3.61; *p* = 0.06; *pƞ*^2^ = 0.02). No other differences between groups were found (see Table 1). The two ANOVAs on the two indices of MAP (day and night) showed significant differences. Considering MAP of daytime (F_2,148_= 3.04; *p*= 0.05; *pn*^2^= 0.04), Extreme Dippers showed higher values than Non-Dippers (F_1,148_ = 5.79; *p* =0.02; *pn*^2^ = 0.04). Regarding the MAP of night-time (F_2,148_ = 42.46; *p* = 0.0001; *pn*^2^ = 0.36), Extreme Dippers showed lower values than both Dippers (F_1,148_ = 23.17; *p* = 0.0001; *pn*^2^ = 0.14) and Non-Dippers (F_1,148_ = 84.89; *p* = 0.0001; *pn*^2^ = 0.36), and Dippers showed lower values than Non-Dippers (F_1,148_ = 28.01; *p* = 0.0001; *pn*^2^ = 0.16) (see Table 1).

No difference in the percentage of men and women in the three groups were highlighted by the χ^2^ analysis (*p* > 20; see Table 1).

### 4.2. Correlations

The correlation matrix is presented in Table 2.

### 4.3. Anger

Table 3 shows the means and standard deviations of the anger scores in the three groups of participants.

ANOVAs on STAXI subscales showed significant differences in Trait Anger Temperament (F_2,148_ = 3.19; *p* = 0.04; *pƞ*^2^ = 0.04), with Extreme Dippers that showed higher scores than both Dippers (F_1,148_ = 4.04; *p* = 0.05; *pƞ*^2^ = 0.03) and Non-Dippers (F_1,148_ = 5.62; *p* = 0.02; *pƞ*^2^ = 0.04); no differences were found between Dippers and Non-Dippers (F_1,148_ = 0.36; *p* = 0.55).

A marginally significant difference between groups was found in Anger Expression-In (F_2,148_ = 2.94; *p* = 0.06; *pƞ*^2^ = 0.04), with Extreme Dippers showing higher scores than both Dippers (F_1,148_ = 4.52; *p* = 0.03; *pƞ*^2^ = 0.03) and Non-Dippers (F_1,148_ = 4.53; *p* = 0.04; *pƞ*^2^ = 0.03); no differences were found between Dippers and Non-Dippers (F_1,148_ < 1; *p* = 0.82).

The ANOVAs did not show other significant differences considering STAXI subscales (see Table 3).

Since age and cigarette consumption were significantly different between groups, they were introduced as covariates in the analyses. 

ANCOVAs showed significant differences between groups in the Anger Expression-In subscale (F_2,146_ = 3.64; *p* = 0.03; *pƞ*^2^ = 0.05), with the Extreme Dippers showing higher scores than both Dippers (F_1,148_ = 5.03; *p* = 0.03; *pƞ*^2^ = 0.03) and Non-Dippers (F_1,148_ = 5.91; *p* = 0.02; *pƞ*^2^ = 0.04).

**Figure 1:**
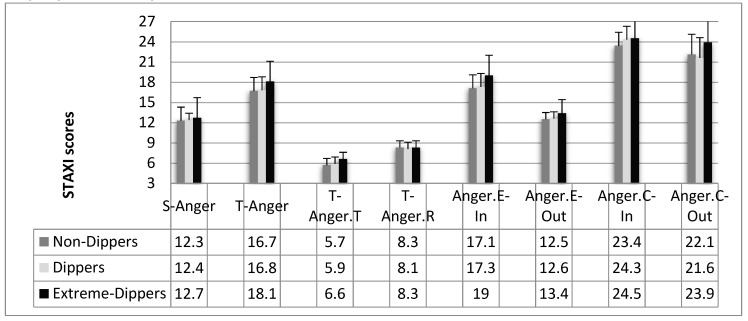
Mean scores and standard errors of the STAXI scales for the three groups of participants.

The ANCOVA on Trait Anger Temperament did not confirm the significant difference (F_2,146_ = 2.51; *p*= 0.09; *pƞ*^2^= 0.03), highlighting a modulatory role of age (F_1,148_ = 8.17; *p* = 0.01; *pƞ*^2^ = 0.06). Planned comparison confirmed only the difference between Extreme Dippers and Dippers (F_1,148_ = 4.07; *p* = 0.04; *pƞ*^2^ = 0.03). Considering the other STAXI subscales, no differences between groups were highlighted (see Figure 1).

### 4.4. Relationship between Anger and the Dipper Phenomenon in Normotensive, Untreated, and Treated Hypertensive Participants

The analyses made did not allow clarifying whether the relationship between anxiety and the dipping phenomenon can vary in normotensive participants and in treated and untreated hypertensive patients because of the low number of participants. However, to probe the influence of the state of blood pressure on the relationship between dipping and anger, nonparametric analyses (χ^2^) were performed. In this case, the Bonferroni correction was applied, which indicated a *p* < 0.02 as significant. The results are shown in Table 4.

These comparisons only showed higher trait anger in Extreme Dippers within normotensive participants and a low trait anger reaction in Extreme Dippers within treated hypertensive patients.

## 5. Discussion

This study aimed to examine the psychological aspects of anger in relation to nocturnal blood pressure dipping and to evaluate the potential mediating roles of confounding variables in this relationship. As hypothesized, the results showed that trait anger was associated with alterations in nocturnal blood pressure dipping. However, this relationship is less clear and linear than we expected, suggesting that only expression of anger is related to extreme dipping of night-time blood pressure independently of other confounding variables. A more complex relationship was highlighted for trait anger, probably due to a larger influence on this personality characteristic of some aspects, such as smoking and aging.

Previous studies observed a relationship of trait anger, anger expression, and anger management with both blood pressure (for a review see [45]) and the dipping phenomenon. However, these results are unclear and contradictory [16,17,36,46,47]. Thomas et al. [31] examined the relationships between dipping, hostility, and anger expression in 86 hypertensive men and women. Individuals who presented higher hostility, anger expression, and anger experience showed lower BP dipping compared to other participants, while participants with greater anger control showed larger BP dipping. Recently, Mezick and colleagues [30] investigated the sleep–wake BP ratios over 48 h in 224 participants. Higher hostility was associated with lower dipping, independently of age, sex, BMI, race, and hypertensive status. Conversely, Linden and colleagues [36] found no association between hostility and BP dipping (as measured by the difference between mean night-time and mean daytime BP) in 62 unmedicated hypertensives. The constructive use of anger [36], as well as its expression, were both associated with higher BP dipping [17]. However, yelling and overtly fighting with others did not affect BP dipping [46]. Similarly, Helmers and colleagues [47] found that inhibiting anger or expressing it on something or someone else had no impact on BP dipping. The heterogeneity of these previous results could be explained by differences in the measurement and classification of BP patterns and anger dimensions. Moreover, inconsistent findings could be due to different samples’ characteristics. We attempted to adopt a well-defined design, a substantial control of confounding variables, and more conservative night and day cut-off times compared to other studies, with the aim to clarify the relationship between anger dimensions and dipping phenomenon.

One of the more interesting results that emerged in the present study is the identification of a higher level of maladaptive anger expression in Extreme Dippers. Our choice to consider the dipping patterns more extensively (i.e., including Extreme Dippers), differently from other studies, allows interpreting the result by taking into account the different characteristics of the dipping patterns associated with cardiovascular risks [48,49,50]. Trait anger temperament and suppression of anger feelings can be associated with high autonomic activation and high hyperarousal during daytime [45]. As a consequence of this pattern, a greater decrease of blood pressure during nighttime, expressed by an Extreme Dipping pattern, could represent a physiological attempt to restore physiological homeostasis.

This assumption is also confirmed by the results about the differences in daytime and night-time MAP between the groups. In fact, Extreme Dippers reported a higher level of daytime MAP and a lower level of nighttime MAP compared to the other groups. People with extreme dipping also had a higher probability of developing a morning surge phenomenon (an excessive increase in blood pressure in the early hours of the morning). Since morning surge is associated with an increased risk of developing stroke and other cardiovascular diseases [51,52], it would be interesting to explore more psychological risk factors associated with this pattern.

Age and smoking have been considered as covariates in the analyses of the relationship between BP dipping and anger, because previous studies demonstrated that they could strongly affect anger, in particular anger expression, and blood pressure [53,54]. The modulatory role of these variables, especially of age, was confirmed. The results underline how the relationship between anger and BP trend during sleep is influenced by age. However, other variables that we did not consider in this study could modulate this relationship. While age reduces the role of trait anger, it appears to strengthen suppression of anger. Aging is associated with an improvement of anger management [55] and with the expression of the non-dipping pattern [56]. Probably, in people who present extreme dipping, this relationship is dissociated, and people express a maladaptive suppression of anger. Generally, this study has highlighted the importance of controlling the role of psychological aspects in the circadian variation of BP and the dipping phenomenon. Previous researches have considered the dipping status as a dichotomous or continuous phenomenon. However, our findings underline the importance of a multidimensional categorical classification. This classification allows considering different forms of nocturnal blood pressure variations such as extreme dipping, which can result in a high risk of cardiovascular diseases [34].

### Limits

Although the present results may add useful information about the dipping phenomenon, this study presents some limits. The small sample size may reduce the effect size of the current results. To extend these findings to the general population, this study should be replicated in larger community samples. Another limit is represented by the use of an ABPM for the assessment of BP. The ABPM is characterized by a poor reproducibility of BP measurements, as it has been highlighted by some authors [57]. However, all precautions were taken to avoid errors, such as using a conservative night range (00:00–06:00) and considering the MAP for dipping classification [40]. Further studies should adopt multiple ABPM measurements over time to control possible environmental and situational artefacts. Given the effect on the dipping pattern of many confounding variables (e.g., age, hypertensive medications, quality of sleep, situational stressor), a single ABPM session could not permit to detect variations of nocturnal BP dipping in response to the change of a psychological aspect, such as anger.

The low percentage of normotensive individuals in our study did not allow us to make inferences on the differences in psychological characteristics between hypertensive and normotensive people with different dipping patterns. Also, the absence of an analysis of drug treatments is a limitation, because we do not know how antihypertensive therapy affected BP circadian variations. It would be useful to analyze this aspect, because it could mediate the relationship between psychological and physiological aspects [25].

Other aspects can be considered both as limitations or as research issues in further studies aimed to analyze the relationship between circadian blood pressure and psychological aspects. First, a limit is the absence of a group of participants with a Reverse Dipping pattern, characterized by an increase of BP in the sleep period [34] and associated with high cardiovascular risk and high mortality [34]. Second, a limit is the absence of an analysis of the morning surge phenomenon (a surge of BP occurring during the early-morning period). An alteration of the BP morning surge appears to be strongly related to cardiac events, such as acute myocardial infarction and ictus [34]; nevertheless, few studies have analyzed its relationship with psychological variables [58,59]. Third, the lack of information on the time of the day of drug treatment for hypertensive participants could have influenced the interpretation of the relationship between anger and the dipping phenomenon. Knowing the timing of anti-hypertensive drugs intake could provide interesting information on the interaction among drugs treatment time, dipping status, and psychological aspects, such as anger. 

## 6. Conclusions

For more than 60 years, many studies have suggested that a higher trait anger or a maladaptive control of anger can represent a cardiovascular risk. This study suggests a pathway by which this emotion and its management may adversely affect cardiovascular functioning. Although knowledge about essential hypertension has increased in the last years, less is known about the circadian rhythm of BP and the dipping phenomenon.

It would be useful to understand these subjects’ characteristics fully and to realize how they interact with other individual characteristics such as psychological dimensions. Increased knowledge of these aspects could improve the diagnosis and treatment of hypertension and related disorders. 

Our findings suggest that psychological factors may contribute to the description and classification of patients who do not exhibit the adaptive dipping phenomenon.

However, although these results are promising, it is necessary to further study the relationship between dipping and psychological characteristics. A more extensive knowledge of these aspects can improve our description of the nature of this relationship and of the causal direction of the variables.

## Figures and Tables

**Table 1:** Means (± SD) of the main characteristics of the three groups of participants.

	Non-Dippers	Dippers	Extreme Dippers	F	*p*
N (Womem/Men)	42 (22/20)	65 (36/29)	44 (26/18)		
Age	60 (8.74)	56 (10.38)	55 (8.76)	3.35	0.04
Years of Education	11.64 (3.33)	12.66 (3.95)	13.41 (4.56)	1.93	0.15
Smoking Cigarettes (number per day)	0.21 (0.57)	0.54 (0.90)	0.27 (0.58)	3.09	0.05
Alcohol’s Consumption (number per day)	0.30 (0.46)	0.37 (0.52)	0.34 (0.52)	0.24	0.79
Body Mass Index (BMI)	27.07 (5.19)	25.81 (4.33)	26.43 (3.87)	1.02	0.36
Systolic Blood Pressure	140.21 (25.73)	142.03 (19.04)	141.70 (14.81)	0.11	0.89
Diastolic Blood Pressure	89.58 (12.88)	91.48 (12.15)	92.63 (9.66)	0.75	0.48
Diurnal Mean Arterial Pressure (MAP)	96.21 (10.59)	97.78 (8.79)	100.86 (7.32)	3.04	0.05
Nocturnal Mean Arterial Pressure Night	90.85 (10.02)	82.77 (7.20)	75.57 (5.61)	42.46	0.0001
Night/Day MAP ratio	0.95 (0.03)	0.85 (0.03)	0.75 (0.04)	416.1	0.0001
Normotensive Participants, *N* (%)	9 (21.4%)	9 (13.8%)	7 (16.0%)		
Hypertensive Untreated Participants, (*N* (%)	7 ^ab^ (16.7%)	21 (32.4%)	20 (45.4%)		
Hypertensive Treated Participants, (*N* (%)	26 ^a^ (61.9%)	35 (53.8%)	17 (38.6%)		

(a) χ^2^ significant differences compared with Extreme Dippers (*p* < 0.05); (b) χ^2^ significant differences compared with Dippers (*p* < 0.05).

**Table 2:** Correlation (Pearson’s r) among dipping ratio, age, cigarette consumption, and anger scores (the *p* values are shown in parentheses).

	(1)	(2)	(3)	(4)	(5)	(6)
**(1) Age**	—					
**(2) Years of Education**	−0.14	—				
**(3) BMI**	0.01	−0.15	—			
**(4) Smoking Cigarettes**	−0.03	−0.04	−0.12	—		
**(5) Alcohol Consumption**	−0.17 *	0.08	−0.02	0.19 *	—	
**(6) Night/Day MAP Ratio**	0.21 **	−0.12	0.05	−0.02	−0.01	—
**(7) T.Anger**	−0.28 ***	−0.14	0.10	0.07	0.05	−0.18 *
**(8) T-Anger.T**	−0.24 **	−0.10	0.03	0.13	0.002	−0.23 **
**(9) T-Anger.R**	−0.19 *	−0.15	0.12	0.004	0.07	−0.05
**(10) Anger.E-In**	−0.17 *	0.19 *	0.03	0.09	−0.07	−0.18 *
**(11) Anger.E-Out**	−0.19 *	−0.11	0.14	0.10	0.02	−0.13
**(12) Anger.C-In**	0.01	0.13	0.175	−0.09	−0.12	−0.04
**(13) Anger.C-Out**	−0.16 *	−0.03	−0.05	0.15	0.05	−0.12

(3) BMI, Body Mass Index; (7) T_Anger, Trait Anger; (8) T-Anger-T, Trait Anger Temperament; (9) T-Anger.R, Trait Anger Reaction; (10) Anger.E-In, Anger Expression In; (11) Anger.E-Out, Anger Expression Out; (12) Anger.C-In, Anger Control In; (13) Anger.C-Out, Anger Control Out. * *p* < 0.05; ** *p* < 0.01; *** *p* < 0.0001.

**Table 3:** Means (± SD) of anger scores and ANOVA results for the three groups of participants.

	Non-Dippers	Dippers	Extreme Dippers	F	*p*
**STAXI**					
**S-Anger**	12.28 (3.92)	12.39 (3.28)	12.70 (4.99)	0.13	0.88
**T-Anger**	16.66 (3.80)	16.78 (4.34)	18.12 (4.96)	1.56	0.21
**T-Anger.T**	5.67 (1.68)	5.89 (1.63)	6.64 (2.39)	3.19	0.04
**T-Anger.R**	8.26 (2.64)	8.12 (2.86)	8.32 (2.83)	0.07	0.93
**Anger.E-In**	17.07 (4.09)	17.26 (3.99)	19.03 (4.81)	2.04	0.05
**Anger.E-Out**	12.52 (2.80)	12.61 (3.11)	13.41 (3.88)	1.12	0.33
**Anger.C-In**	23.44 (6.26)	24.33 (5.53)	24.51 (5.35)	0.44	0.64
**Anger.C-Out**	22.15 (7.94)	21.55 (7.74)	23.93 (8.92)	1.15	0.32

S-Anger, State Anger; STAXI, State–Trait Anger Expression Inventory.

**Table 4:** Means of anger scores and χ^2^ results for Non-Dippers, Dippers, and Extreme Dippers in the three groups of Normotensive, Untreated, and Treated Hypertensive participants.

	Non-Dippers	Dippers	Extreme Dippers	*χ^2^*	*p*
*Normotensive Participants*					
**S-Anger**	11.22 (1.30)	12.89 (3.02)	14.57 (9.16)	<1	0.63
**T-Anger**	16.00 (3.64)	13.67 (1.66)	20.42 (6.26)	6.08	0.04
**T-Anger.T**	5.67 (1.66)	5.00 (0.50)	8.00 (2.58)	8.89	0.01
**T-Anger.R**	7.33 (1.87)	5.89 (1.36)	9.28 (2.98)	6.08	0.04
**Anger.E-In**	16.00 (4.06)	18.00 (2.34)	19.14 (5.39)	1.21	0.54
**Anger.E-Out**	13.44 (3.24)	12.67 (2.18)	1.85 (4.29)	1.16	0.55
**Anger.C-In**	21.13 (6.73)	24.52 (4.44)	24.34 (3.54)	2.11	0.34
**Anger.C-Out**	24.32 (8.26)	22.14 (6.10)	26.65 (10.17)	2.36	0.31
*Hypertensive Untreated Participants*					
**S-Anger**	12.28 (3.14)	12.09 (3.59)	12.06 (2.58)	<1	0.73
**T-Anger**	18.14 (5.58)	16.61 (4.62)	18.87 (5.38)	4.27	0.11
**T-Anger.T**	6.57 (2.63)	6.19 (1.43)	6.70 (2.45)	<1	0.98
**T-Anger.R**	8.57 (3.31)	7.76 (3.19)	8.65 (3.01)	4.41	0.11
**Anger.E-In**	15.29 (1.60)	16.46 (3.58)	19.38 (5.26)	5.42	0.06
**Anger.E-Out**	12.43 (3.05)	13.57 (3.66)	13.30 (3.76)	<1	0.68
**Anger.C-In**	21.28 (8.09)	23.17 (5.21)	24.05 (6.43)	<1	0.82
**Anger.C-Out**	22.43 (11.76)	22.85 (7.91)	24.63 (8.66)	<1	0.90
*Hypertensive Treated Participants*					
**S-Anger**	12.65 (4.66)	12.43 (3.21)	12.71 (5.05)	2.45	0.29
**T-Anger**	16.49 (3.34)	17.68 (4.33)	16.29 (3.22)	1.19	0.54
**T-Anger.T**	5.42 (1.33)	5.94 (1.86)	6.00 (2.12)	<1	0.88
**T-Anger.R**	8.50 (2.70)	8.91 (2.68)	7.52 (2.48)	7.24	0.02
**Anger.E-In**	17.92 (4.43)	17.55 (4.54)	18.59 (4.26)	<1	0.94
**Anger.E-Out**	12.23 (2.63)	12.03 (1.87)	12.53 (3.64)	<1	0.84
**Anger.C-In**	24.83 (5.37)	24.97 (5.96)	25.11 (4.74)	1.77	0.41
**Anger.C-Out**	21.32 (6.79)	20.61 (8.07)	22.00 (8.86)	<1	0.87
